# Increasing G9a automethylation sensitizes B acute lymphoblastic leukemia cells to glucocorticoid-induced death

**DOI:** 10.1038/s41419-018-1110-z

**Published:** 2018-10-10

**Authors:** Coralie Poulard, Estelle Baulu, Brian H. Lee, Miles A. Pufall, Michael R. Stallcup

**Affiliations:** 1 Department of Biochemistry and Molecular Medicine, Norris Comprehensive Cancer Center, University of Southern California, Los Angeles, CA 90089 USA; 2 Department of Biochemistry, Holden Comprehensive Cancer Center, University of Iowa, Iowa City, IA 52242 USA

## Abstract

Synthetic glucocorticoids (GCs) are used to treat lymphoid cancers, but many patients develop resistance to treatment, especially to GC. By identifying genes that influence sensitivity to GC-induced cell death, we found that histone methyltransferases G9a and G9a-like protein (GLP), two glucocorticoid receptor (GR) coactivators, are required for GC-induced cell death in acute lymphoblastic leukemia (B-ALL) cell line Nalm6. We previously established in a few selected genes that automethylated G9a and GLP recruit heterochromatin protein 1γ (HP1γ) as another required coactivator. Here, we used a genome-wide analysis to show that HP1γ is selectively required for GC-regulated expression of the great majority of GR target genes that require G9a and GLP. To further address the importance of G9a and GLP methylation in this process and in cell physiology, we found that JIB-04, a selective JmjC family lysine demethylase inhibitor, increased G9a methylation and thereby increased G9a binding to HP1γ. This led to increased expression of GR target genes regulated by G9a, GLP and HP1γ and enhanced Nalm6 cell death. Finally, the KDM4 lysine demethylase subfamily demethylates G9a in vitro, in contrast to other KDM enzymes tested. Thus, inhibiting G9a/GLP demethylation potentially represents a novel method to restore sensitivity of treatment-resistant B-ALL tumors to GC-induced cell death.

## Introduction

Acute lymphoblastic leukemia (ALL) is the most common cancer of childhood, representing 30% of all childhood cancers and 80% of childhood leukemias. Treatment consists of a combination of chemotherapeutic agents, including vincristine, L-asparaginase and synthetic glucocorticoid (GC) agonists, such as dexamethasone (dex) and prednisolone^1^. With recent progress in ALL therapy, the 5-year survival rate now approaches 90%^2^. Nevertheless, about 10–20% of children with ALL do not respond to combination chemotherapy that includes GC, or they develop resistance upon relapse; this treatment resistance is strongly correlated with GC insensitivity^2–4^. Adverse side effects, including osteoporosis, hyperglycemia, hyperlipidemia, insulin resistance, muscle wasting and obesity, are frequently associated with long-term, high-dose GC treatments, such that an increased number of patients experience life-threatening morbidity by their 30s, including heart and lung disease, secondary cancers and developmental problems^5,6^. Thus, novel treatments based on an enhanced understanding of GC-induced cell death and mechanisms of resistance are clearly needed.

The natural human GC is cortisol, a steroid hormone that regulates numerous physiological functions and plays an important role in response to stress, countering inflammation, and maintenance and reestablishment of metabolic homeostasis. The powerful anti-inflammatory and immune suppressive actions of GC are mechanistically broad-based and complex, but include their pro-apoptotic effect on lymphocytes, which is relevant to their wide-spread use in treatment of many types of blood cancer^7^. GCs activate the glucocorticoid receptor (GR), which activates and represses specific genes. GR binds specific gene regulatory elements in DNA and recruits coregulators which modulate local chromatin conformation and regulate formation of active transcription complexes on neighboring gene promoter sites^8^. Coregulator actions are gene specific, i.e., each coregulator is required for only a subset of genes regulated by GR^9–13^. Thus, while GCs regulate many physiological pathways, specific coregulators are preferentially required for GC regulation of genes involved in selected GC physiological responses^12–14^. Therefore, if coregulators involved in GC regulation of the apoptosis pathway can be identified, the gene-specific nature of coregulator function may make them useful targets for selective enhancement of GC action in treatment of relapsed lymphoid cell-derived cancers while minimizing GC side effects.

Starting with a genome-wide short hairpin RNA (shRNA) screen, we recently demonstrated that coregulators G9a (EHMT2) and G9a-like protein (GLP; EHMT1) are required for efficient GC-induced apoptosis of the Nalm6 B-ALL cell line^15^. G9a and GLP are highly homologous lysine methyltransferases that serve as coactivators for some GR target genes and corepressors for others, while a third larger group of GR target genes is regulated by GC independently of G9a and GLP^13^. We showed in A549 lung adenocarcinoma cells^13^ that adjacent N-terminal methylation and phosphorylation of G9a and GLP oppositely regulate the coactivator function. Automethylated G9a and GLP recruit heterochromatin protein 1γ (HP1γ) which helps to recruit RNA polymerase II to begin transcription of GR target genes, but phosphorylation of the threonine residue adjacent to the methylation site by Aurora kinase B (AurkB) prevents HP1γ binding to G9a and GLP and thus inhibits their coactivator function^13^.

As G9a/GLP automethylation is required to recruit HP1γ as a requisite component of G9a/GLP coactivator function, we hypothesized that increasing the level of the methylation modification on G9a/GLP could increase sensitivity of the B-ALL cells to GC-induced cell death. Indeed, lysine methylation and demethylation of proteins are now known to be dynamic processes, so that inhibiting demethylation of G9a and GLP should in principle enhance their methylation status. There are two families of lysine demethylases (KDM), the lysine-specific demethylase (LSD) family and the jumonji C (JmjC) family^16^. The two LSD family members are amine oxidases which demethylate mono- and dimethyllysine residues in a flavin adenine dinucleotide-dependent manner. The JmjC family is composed, so far, of 18 JmjC domain-containing proteins which demethylate mono-, di- and trimethyllysine residues via a dioxygenase reaction requiring Fe(II) and α-ketoglutarate as cofactors^17^. Although KDMs have been primarily studied in connection with histone demethylation, they also demethylate non-histone substrates^18^. To test our hypothesis that increased G9a/GLP methylation would enhance GC-induced apoptosis of B-ALL cells, we employed inhibitors that target each KDM family to find one that increased methylation levels of G9a. To facilitate mechanistic studies, we used cells depleted of G9a, GLP or HP1γ to identify the specific GC-regulated genes that required these proteins as coactivators. The inhibitors were then tested for their ability to enhance the GC-induced expression of the G9a/GLP/HP1γ-dependent genes, the interaction of G9a and GLP with HP1γ and GC-induced apoptosis of the Nalm6 B-ALL cell line.

## Materials and methods

### Cell culture

Nalm6 cells were purchased from American Type Culture Collection and maintained in RPMI-1640 medium containing l-glutamine and 10% fetal bovine serum at 37 °C and 5% CO_2_. CV-1, 293T and A549 cells were purchased from American Type Culture Collection and maintained in Dulbecco’s modified Eagle’s medium supplemented with 10% fetal bovine serum at 37 °C and 5% CO_2_.

For JIB-04 (Tocris) and OG-L002 (Selleckchem) inhibitors, cells were treated with the indicated concentration or with equivalent volume of dimethyl sulfoxide (DMSO) for the indicated time. When indicated, cells were treated with 100 nM of dex (Sigma) or an equivalent amount of vehicle ethanol for the indicated time.

For lentivirus particle production, 293T cells were plated in 100 mm dishes and transiently transfected by Lipofectamine 3000 (Invitrogen) according to the manufacturer’s protocol with the lentiviral vector (pMK1221-shG9a, pMK1221-shGLP or pMK1221-shHP1γ) and the 3rd-generation lentiviral packaging constructs (VSV-G, RSV, MDL, Addgene nos. 12259, 12253, and 12251, respectively). The viruses were harvested by collecting the medium at 24 and 48 h post transfection. Virus-containing medium from two harvests was pooled, passed through a 0.45 μm filter and stored at −80 °C. For lentiviral transduction, 5 million Nalm6 cells were incubated with virus-containing medium and polybrene (Millipore) at the final concentration of 8 μg/ml for 5 min. Then, the mixture was transferred to 6-well plates and cells were spinfected for 2 h at 1000 × *g* and 33 °C. After spinning, virus-containing medium was removed, and cells resuspended in fresh medium. At 48 h after infection, puromycin was added (2 μg/ml) for selection of infected cells. After 72 h of selection, the resistant cell populations were used for the indicated experiments.

### Plasmids

The following plasmids were described previously: luciferase reporter plasmid MMTV-LUC (which contains glucocorticoid responsive elements), along with mammalian protein expression vectors for hG9a FL WT and hG9a FL K/R, hGR and mGrip1 and bacterial expression vectors for GST-hGLP N (amino acids 31–357) and GST-hGLP ΔN (amino acids 814–1279)^13,19,20^. pCMV-HA-KDM4A (#24180), pCMV-HA-KDM4B (#24181) and pCMV-HA-KDM4C (#24214) were obtained from Addgene.

### Quantitative real-time reverse transcription PCR (RT-qPCR)

RNA was isolated using TRIzol (Invitrogen) according to the manufacturer’s instructions. Reverse transcription reaction was performed using Superscript III (ThermoFisher) according to specifications with 1 μg of total RNA as template. Quantitative PCR amplification of the resulting complementary DNA (cDNA) was performed on a Roche LightCycler 480 using SYBR green I master mix (Roche). Messenger RNA (mRNA) levels were normalized to the level of β-actin mRNA. The following primers were used to detect mRNA. TSC22D3 (forward primer, 5′-GCTGTGAGAGAGGAGGTGGA-3′; reverse primer, 5′-CTTCAGGGCTCAGACAGGAC-3′), TXNIP (forward primer, 5′-GCCACACTTACCTTGCCAAT-3′; reverse primer, 5′-GTTGCAGCCCAGGATAGAAG-3′), LILRA2 (forward primer, 5′-GGAAGTCCTGTGACCCTCAG-3′; reverse primer, 5′-GTGTTCCCAGGTGATGGATG-3′), LDLRAD4 (forward primer, 5′-AGTTCGCCCAAATCATCATC-3′; reverse primer, 5′-GGGCGGTTGATGAAGGAC-3′), ARHGAP6 (forward primer, 5′-CCTCTTGCCCATCTTTGCT-3′; reverse primer, 5′-TGTTGGAGCCGGAGGAAC-3′), ZNF831 (forward primer, 5′-AAAGGGAATTTCTTGCAGAGC-3’; reverse primer, 5′-CATGGCTTCAGTCCCTCAA-3′), β-actin (forward primer, 5′-CCACACTGTGCCCATCTACG-3′; reverse primer, 5′-AGGATCTTCATGAGGTAGTCAGTCAG-3′).

### RNA-sequencing analysis

Quality of the cDNA was assessed using Agilent Technologies 2100 Bioanalyzer. A total of 24 high-quality samples (8 conditions × 3 replicates each) were submitted to DNA Link, Inc. for library preparation and sequencing. Illumina NEXTseq 500 was used to generate paired-end 75 bp RNA-sequencing data for all the samples. Sequencing results produced 37–48 million raw reads per sample. RNA-sequencing data have been submitted to Gene Expression Omnibus (GEO) (GSE accession number GSE117796). After trimming the raw reads for quality and adapter sequence, the samples were mapped using HISAT2 against the hg19 human reference genome^21^. Mapped reads were quantified to known RefSeq genes using the GenomicAlignments R package^22^. Gene expression levels were normalized with the upper quantile method and lowly expressing genes were excluded such that genes with more than one count per million in at least two samples were analyzed^23^. We implemented the remove unwanted variation strategy to account for batch effects between samples^24^. Differentially expressed genes were identified with edgeR using a fold change expression cutoff where indicated and false discovery rate adjusted *p* value ≤ 0.05^25^. Box plots were generated using R. The horizontal center lines indicate the median with the upper and lower box limits denoting the 25th and 75th percentile, respectively. The whiskers extend 1.5 times the interquartile range from the 25th and 75th percentiles. The *p* values indicating statistical significance between the datasets in the box plots were obtained using the Mann–Whitney *U* test.

### Immunoprecipitation and immunoblot

Cell extracts were prepared in RIPA buffer (50 nM Tris-HCl, pH 8, 150 mM NaCl, 1 mM EDTA, 1% NP-40 and 0.25% deoxycholate) supplemented with protease inhibitor tablets (Roche Molecular Biochemicals) and phosphatase inhibitors (1 mM NaF, 1 mM Na_3_VO_4_, and 1 mM β-glycerophosphate). Protein extracts were incubated overnight at 4 °C with shaking with 1 μg of antibodies against HA epitope (Roche Applied Science 3F10) or pan methyllysine (AbCam ab-23366). Protein A/G plus agarose beads (Santa Cruz sc-2003) were added, and the mixture was incubated for 2 h at 4 °C. The immunoprecipitates were separated on sodium dodecyl sulfate–polyacrylamide gel electrophoresis. Immunoblots were performed with primary antibodies against G9a (Sigma G6919), β-actin (Sigma A5441), GLP (Millipore 09-078), HP1γ (Abcam ab10480), cleaved Caspase 3 (Cell Signaling 9664S), cleaved Caspase 7 (Cell Signaling 8438S), cleaved PARP1 (Cell Signaling 9541S), HA epitope (3F10 Roche Applied Science), H3K9me2 (Cell signaling 4858S), histone H3 (Cell signaling 4499), GAPDH (Sigma G9545) or Flag (Sigma F1804). Secondary antibodies from Promega were used for chemiluminescence detection using ECL prime detection reagent (Amersham) according to the manufacturers’ instructions.

### Cell death assays

Cells were plated in CellStar low evaporation lid 96-well round-bottom plate at a density of 250,000 cells/ml. Cells were treated directly after plating in triplicate with serial dilutions of dexamethasone or vehicle control (0.1% ethanol) plus other additions as indicated. After 72 h, cell viability of each well was analyzed in duplicate using the Presto Blue Assay Reagent (Life Technologies). Fluorescence was measured, and data were analyzed using Prism6 software.

### Luciferase assays

CV-1 cells were plated in 24-well plates the day before transfection. Cells were transfected using Lipofectamine 2000 (Invitrogen) with the indicated plasmids according to the manufacturer’s protocol. After transfection, the cells were grown in hormone-free medium with 5% charcoal-stripped serum for 48 h in the presence or absence of 100 nM dex. Cell lysis and luciferase assays on cell extracts were performed with Promega luciferase assay kit.

### Cell migration

A549 cells suspended in serum-free medium were plated in the upper part of a 24-well, 8 μm pore, Transwell cell migration chamber (Cell Biolabs Inc, San Diego, CA, USA) according to the manufacturer’s protocol as previously described^13^.

### Proximity ligation assays

The experiments were performed following the manufacturer’s instructions as previously described^26,27^. The samples were analyzed on Zeiss Imager.Z1 fluorescence microscope. For each sample, interactions were counted for 1000 cells using ImageJ software^28^.

### In vitro methylation and demethylation assays

For G9a methylation 1 μg of recombinant G9a protein (hG9a FL, Active Motif, #31410) was incubated with 1 mM unradiolabeled *S*-adenosylmethionine (SAM, New England Biolabs, B9003S) for 3 h at room temperature in methylation buffer (50 mM Tris-HCl pH 8.6, 0.02% Triton X-100, 2 mM MgCl_2_, 1 mM TCEP (tris(2-carboxyethyl)phosphine)). For GLP methylation bacterially produced glutathione *S*-transferase (GST) fusion proteins (2 μg) of N-terminal fragments of GLP (GST-hGLP N) were incubated for 90 min at 30 °C with GST-hGLP ΔN in the presence of 1 mM of unradiolabeled SAM.

For in vitro demethylation assay, previously methylated hG9a FL or GST-hGLP N were separated from residual SAM using the Amicon Ultra Centrifugal Filter Units (Millipore). Then, methylated product was incubated with the following recombinant protein demethylases from Active Motif: JMJD1A/KDM3A (#31456), JMJD2A/KDM4A (#31457), JMJD2B/KDM4B (#31501), JMJD2C/KDM4C (#31458), JARID1A/KDM5A (#31431) or JMJD3/KDM6B (#31519) or GST as a control for 3 h at room temperature in demethylation buffer (50 mM Hepes pH 7.5, 0.02% Triton X-100, 100 μM 2-Oxoglutarate, 100 μM Ascorbate, 50 μM (NH_4_)_2_Fe(SO_4_)_2_·6H_2_O, 1 mM TCEP). Methylation status was analyzed by immunoblot using pan methyllysine antibody (ab-23366, Abcam). Then, the polyvinylidene difluoride membrane was incubated in a Coomassie brilliant blue staining solution and destained to observe the recombinant proteins.

## Results

### HP1γ is required for G9a/GLP coactivator function

**Fig. 1:**
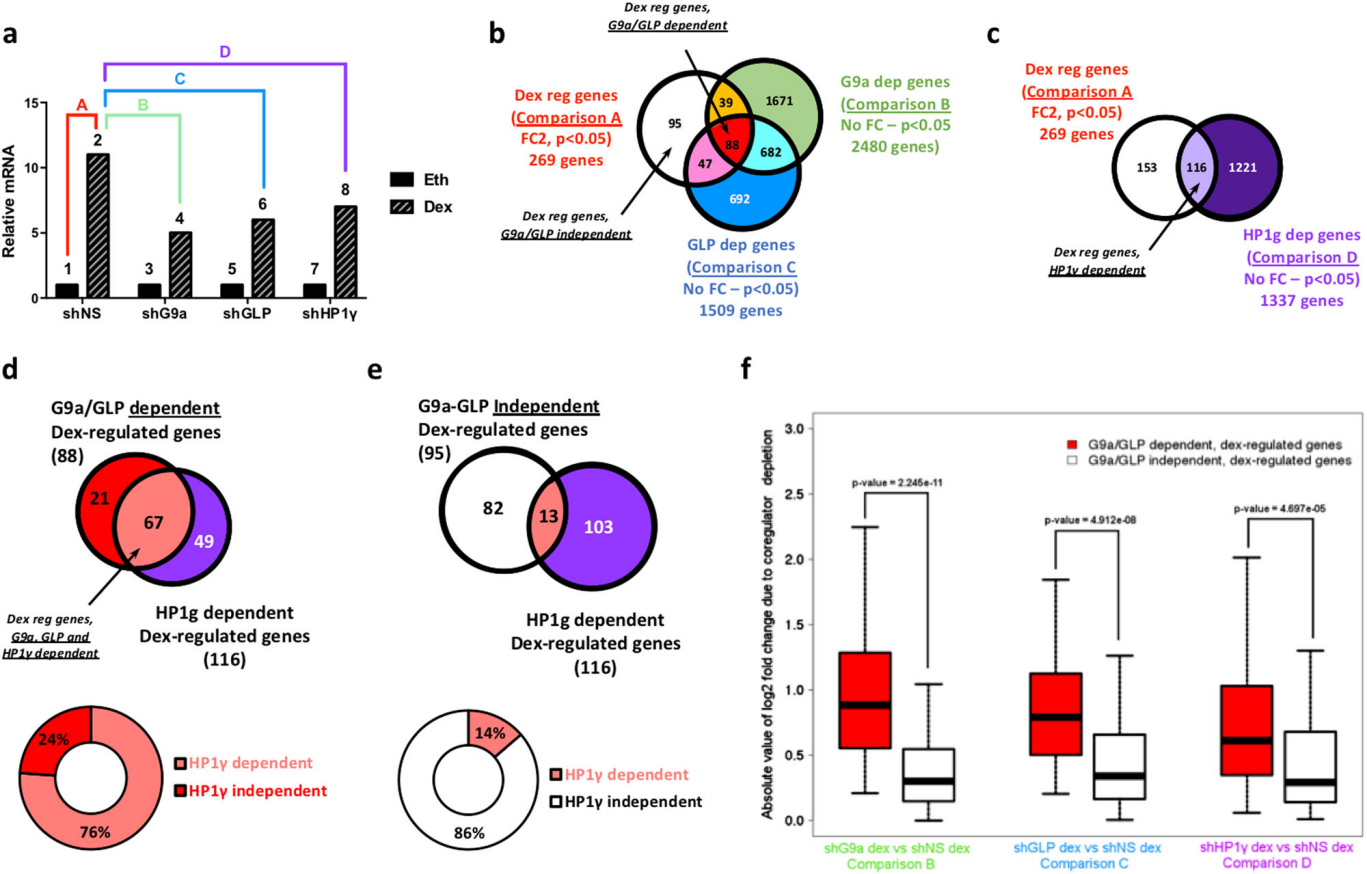
HP1γ is required for expression of most dex-regulated genes that are G9a/GLP dependent. Genome-wide RNA-sequencing analysis was performed on Nalm6 cells to identify the genes dependent on G9a, GLP and HP1γ for dex-regulated expression. **a** Hypothetical results of gene expression profiles for a given gene illustrate how specific pairwise comparisons between datasets for individual samples were performed. Numbered bars represent hypothetical mRNA levels from RNA-seq data for cells expressing the indicated shRNA and treated for 8 h with ethanol (Eth) or 100 nM dex. Colored letters represent pairwise comparisons performed to determine sets of genes for which the mRNA levels were significantly different between the bracketed cell samples: i.e., comparison A = set of dex-regulated genes (fold change ≥2, adjusted *p* ≤ 0.05); comparison B = set of G9a-dependent genes (no fold change cutoff, adjusted *p* ≤ 0.05); C = set of GLP-dependent genes (no fold change cutoff, adjusted *p* ≤ 0.05); D = set of HP1γ-dependent genes (no fold change cutoff, adjusted *p* ≤ 0.05). **b** White Venn diagram represents the dex-regulated genes in cells expressing shNS (comparison A); green Venn diagram, G9a-dependent genes in dex-treated cells (comparison B); blue Venn diagram, GLP-dependent genes in dex-treated cells (comparison C). Overlap areas indicate the number of genes shared among sets. **c** White Venn diagram represents the dex-regulated genes in cells expressing shNS (comparison A); purple Venn diagram, HP1γ-dependent genes in dex-treated cells (comparison D). Overlap areas indicate the number of genes shared among sets. **d** Red Venn diagram represents the dex-regulated genes that are dependent on both G9a and GLP, identified in the red overlap area of (**b**); purple Venn diagram, dex-regulated genes that are HP1γ dependent, identified in the light purple overlap area of (**c**). The percentages of dex-regulated, G9a/GLP-dependent genes that are HP1γ dependent or HP1γ independent are represented in the donut graph. **e** White Venn diagram represents the dex-regulated genes that are independent of both G9a and GLP, identified in the white sector of (**b**); purple Venn diagram, dex-regulated genes that are HP1γ dependent, identified in the light purple overlap area of (**c**). The percentages of dex-regulated, G9a/GLP-independent genes that are HP1γ dependent or HP1γ independent are represented in the donut graph. **f** Boxplot of the absolute value of log_2_ fold change in mRNA levels due to depletion of G9a, GLP or HP1γ. Gene expression changes caused by depletion of G9a, GLP, and HP1γ in dex-treated cells are shown for all dex-regulated genes that are G9a/GLP dependent (red) and G9a/GLP independent (white)

Because we previously found that HP1γ is selectively required for GC-induced expression of several genes that require G9a and GLP as coactivators^13^, we employed RNA interference and RNA-sequencing to explore whether this association applies genome-wide. RNAs were prepared from the B-ALL cell line Nalm6 expressing shRNA against G9a, GLP, HP1γ or a non-specific sequence (shNS) and treated with either 100 nM dex or vehicle ethanol for 8 h. In three biological replicates G9a, GLP and HP1γ protein and mRNA were efficiently depleted by the relevant shRNA (Fig. S1a-b). We identified 269 genes for which the mRNA level was changed significantly and by at least 2-fold in the shNS cells after 8 h of dex treatment (Fig. 1a, comparison A; Fig. 1b, c, white circle). We then identified the subset of dex-regulated genes that require G9a and GLP for dex-regulated expression. Because the dex-induced cell phenotype is determined more by the levels of gene products after dex treatment than by the dex-induced fold change, we analyzed the effect of G9a and GLP depletion by comparing gene expression profiles in the dex-treated cells expressing shNS versus shG9a (Fig. 1a, comparison B; Fig. 1b, green circle) or shGLP (Fig. 1a, comparison C; Fig. 1b, blue circle). For determining genes that are G9a or GLP dependent, differentially expressed genes are defined as those with a significant *p* value (*p* < 0.05) and no fold change cutoff, in order to maximize the number of genes included and thus provide more statistical power for subsequent analysis. By overlapping the sets of genes identified in comparisons A, B and C, we identified 88 genes common to all three sets (Fig. 1b, central red overlap). We classified these genes as dex regulated and G9a/GLP dependent. A similar analysis identified 1337 HP1γ-dependent genes by comparing gene expression profiles in the dex-treated cells expressing shNS versus shHP1γ (Fig. 1a, comparison D; Fig. 1c, purple circle). The overlap between HP1γ-regulated genes and the dex-regulated genes gave us the genes that are dex regulated and HP1γ dependent (Fig. 1c, light purple overlap). The comparison between the 88 dex-regulated, G9a/GLP-dependent genes (Fig. 1b) and the 116 dex-regulated, HP1γ-dependent genes (Fig. 1c) gave us 67 dex-regulated genes that are dependent on G9a, GLP and HP1γ (Fig. 1d, pink overlap). Overall, 76% (67 of 88) of the dex-regulated genes that require G9a and GLP also require HP1γ for their dex-regulated expression (Fig. 1d, lower diagram). In contrast, only 13 of the 95 dex-regulated genes that are independent of G9a and GLP (14%) require HP1γ for their dex-regulated expression (Fig. 1e). A similar analysis with G9a-, GLP- or HP1γ-dependent genes defined by a more stringent 1.3-fold change caused by coregulator depletion produced similar results, confirming our conclusions (Fig. S2).

For a more quantitative analysis of the global effect of coregulator depletion, we analyzed the absolute change due to HP1γ depletion of the mRNA levels in dex-treated cells for all dex-regulated, G9a/GLP-dependent genes and for all dex-regulated, G9a/GLP-independent genes (Fig. 1f). The absolute changes in mRNA levels due to HP1γ depletion were significantly greater for the G9a/GLP-dependent, dex-regulated genes in comparison with the G9a/GLP-independent, dex-regulated genes (Fig. 1f, right diagram), indicating that HP1γ is preferentially required for the same subset of dex-regulated genes that require G9a and GLP. To compare these results to a positive control, the same type of analysis for G9a depletion and GLP depletion produced results similar to those for the HP1γ depletion (Fig. 1f, left and middle diagrams).

**Fig. 2:**
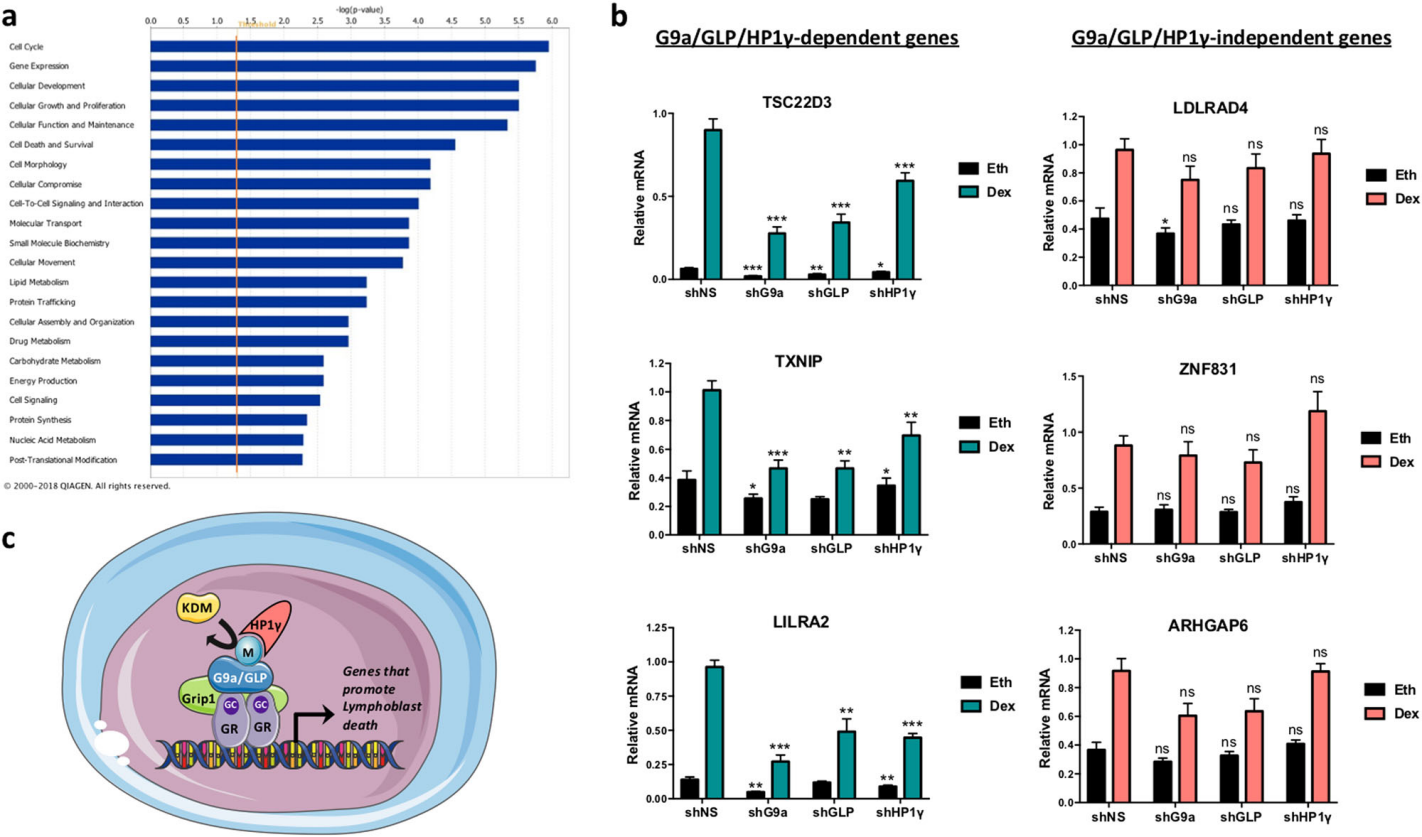
Analysis of dex-regulated genes that are dependent or independent of G9a, GLP and HP1γ. **a** Ingenuity pathway analysis of cellular functions for the 67 dex-regulated genes that are dependent on G9a, GLP and HP1γ (from Fig. 1d). The threshold for significance is indicated by the vertical orange line. **b** NALM-6 cells expressing shRNA against G9a, GLP, HP1γ or a non-specific sequence (shNS) were treated for 8 h with 100 nM dex or equivalent volume of vehicle ethanol (Eth). mRNA levels for the indicated G9a/GLP/HP1γ-dependent genes (left side, chosen from the 67 genes in the overlap of Fig. 1d) and the indicated G9a/GLP/HP1γ-independent genes (right side, chosen from the 82 genes in the white sector of Fig. 1e) were measured by RT-qPCR and normalized to β-actin mRNA levels. Results shown are mean ± SEM for six independent experiments. The *p* value was calculated using a paired *t*-test comparing results for dex-treated cells expressing shRNA against G9a, GLP or HP1γ to the dex-treated shNS sample, *p ≤ 0.05, **p ≤ 0.01, ***p ≤ 0.001, ns not significant. **c** Model for regulation of lymphoblast death in B-ALL cells. Methylated G9a or GLP recruit HP1γ which facilitates the recruitment of RNA polymerase II and activates the expression of genes involved in cell death. G9a and GLP methylation is removed by a specific demethylase (KDM). Theoretically, inhibiting this KDM would increase the methylation and subsequently the GC-induced cell death

A pathway analysis of the 67 genes that require G9a, GLP and HP1γ for their dex-regulated expression level indicated that genes involved in cell cycle, cellular growth and proliferation, and cell death and survival were enriched (Fig. 2a, Table S1), consistent with the role of these three coregulators in facilitating dex-induced death of Nalm6 cells. Among the subset of dex-induced genes that require G9a, GLP and HP1γ, we identified TXNIP and TSC22D3 (aka GILZ), two master genes of GC-induced apoptosis^29–31^ (Table S1). Dex-induced expression of TXNIP contributes to an increase in oxidative stress, whereas dex induction of TSC22D3 inactivates nuclear factor-κB and activator protein-1 (AP-1) survival pathways^29,32,33^.

As validation of the RNA-seq results, quantitative RT-PCR confirmed that depletion of G9a, GLP or HP1γ by shRNAs significantly decreased dex-induced expression levels of TXNIP, TSC22D3 and LILRA2, which were identified as G9a, GLP and HP1γ dependent in the RNA-seq analysis (Fig. 2b, left). In contrast, depleting these coregulators had no effect on dex-induced expression of genes identified as G9a/GLP/HP1γ independent in the RNA-seq analysis (Fig. 2b, right).

### Inhibition of a subset of KDMs sensitizes Nalm6 cells to GC-induced death

**Fig. 3:**
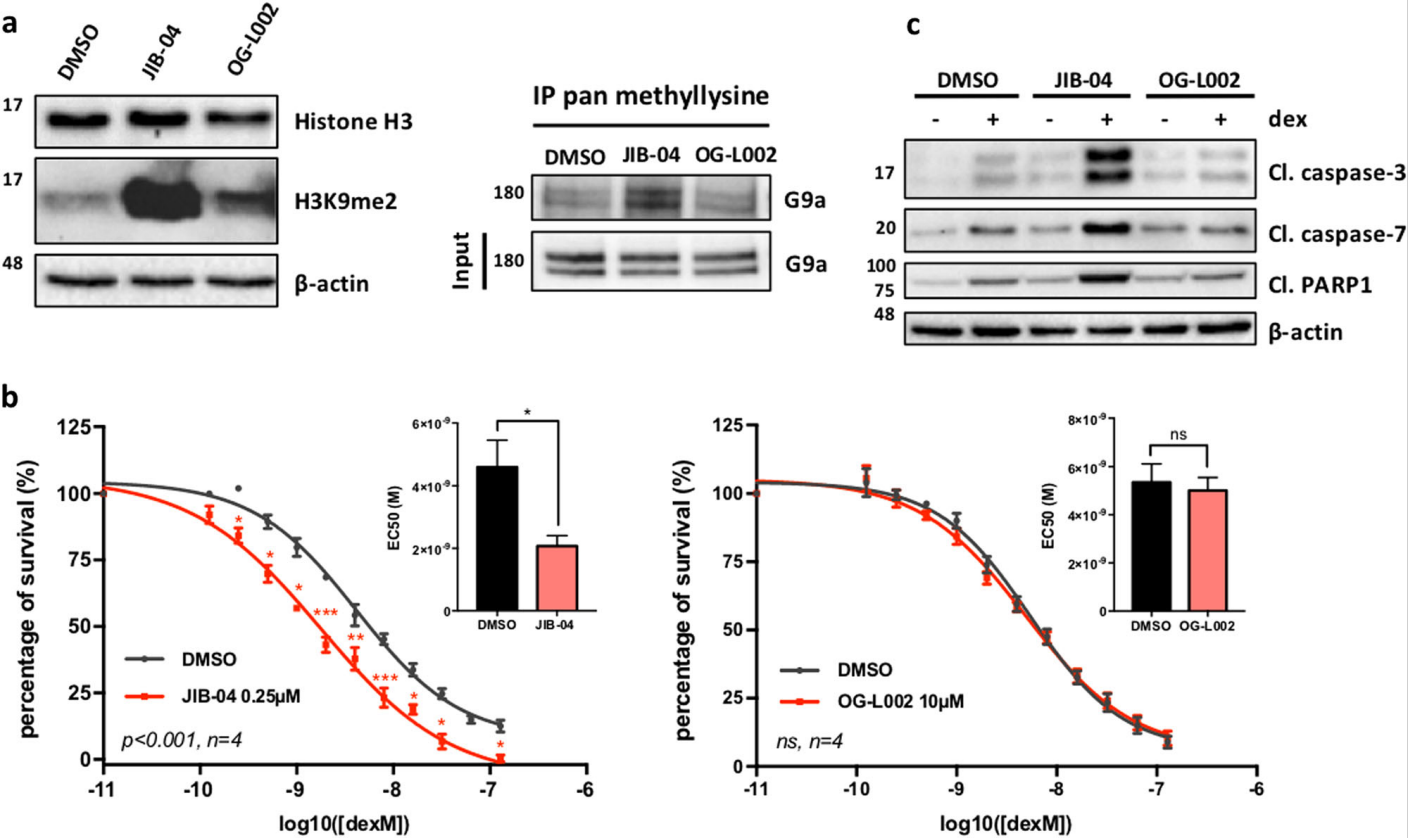
JIB-04 demethylase inhibitor sensitizes B-ALL cells to dex-induced cell death. **a** Nalm6 cells were treated with JIB-04 at 0.5 μM, OG-L002 at 10 μM or vehicle DMSO for 24 h. Lysates were analyzed by immunoblot with the indicated antibodies (left panel) or immunoprecipitated with pan methyllysine antibody and analyzed by immunoblot with G9a antibody (right panel). Results shown are representative of three independent experiments. **b** Nalm6 cells were treated with vehicle DMSO or with JIB-04 at 0.25 μM (left panel) or OG-L002 at 10 μM (right panel) for 72 h in addition to a serial dilution of dex, and cell survival was measured by fluorescence metabolic assay. The fluorescence intensity for dex-treated cells was normalized to that measured with ethanol-treated cells. Percentage of survival is shown as the mean ± SEM for 4 independent experiments (each performed with triplicate biological replicates) and *p* values for results at individual dex concentrations were calculated using a paired *t*-test; *p ≤ 0.05, **p ≤ 0.01, ***p ≤ 0.001. A *F*-test was also calculated to compare the two curves. Insets show the corresponding half-maximal effective concentration (EC_50_) values. **c** Nalm6 cells were first treated with JIB-04 at 0.5 μM, OG-L002 at 10 μM or DMSO for 4 h, then 100 nM of dex or ethanol was added to the media for an additional 20 h. Lysates were analyzed by immunoblot with the indicated antibodies. Results shown are representative of three independent experiments

Since automethylation of the G9a/GLP N-terminal domain is required for their coactivator activity^13^, we hypothesized that increasing the methylation level of G9a/GLP by inhibition of the relevant KDM(s) would enhance the coactivator function of G9a/GLP (Fig. 2c). To test this, we employed two class-specific KDM inhibitors, LSD family inhibitor OG-L002^34^ and JIB-04 which inhibits several members of the JmjC family of KDMs^35^. To confirm the efficacy of JIB-04 and OG-L002 inhibitors in Nalm6 cells, we analyzed histone H3 dimethylation at lysine 9 (H3K9me2), which is a substrate for KDMs that are inhibited by both JIB-04 and OG-L002. Treatment of Nalm6 cells with JIB-04 or OG-L002 increased H3K9me2 level compared to the control (DMSO-treated) cells (Fig. 3a, left panels), with JIB-04 causing a much more dramatic increase than OG-002. Next, we examined the methylation level of G9a in Nalm6 cells by immunoprecipitation with pan methyllysine antibody followed by immunoblot with antibody against G9a. JIB-04 increased G9a methylation compared to vehicle control DMSO, but OG-L002 had no effect (Fig. 3a, right panels). Thus, JIB-04 inhibits one or more KDMs that target G9a.

According to our model, increasing G9a methylation should enhance GC-induced expression of genes that are G9a/GLP dependent, which includes genes that contribute to GC-induced cell death (Fig. 2c). To test this model, Nalm6 cells were treated with JIB-04, OG-L002 or vehicle DMSO as a control, along with serial dilutions of dex for 72 h, and cell viability was analyzed. JIB-04 treatment increased the sensitivity of the cells to dex, compared with the DMSO control, but OG-L002 had no effect on GC-induced cell death (Fig. 3b). JIB-04 alone reduced cell viability to 70%, but in the figure we normalized all of the data from JIB-04-treated cells so that the viability with JIB-04 alone was 100% in order to observe better the effect on dex sensitivity. JIB-04, but not OG-L002, also enhanced dex-induced cleavage of three apoptosis markers (Fig. 3c). JIB-04 alone and OG-L002 alone caused slight increases in cleavage, presumably due to some cell toxicity. However, co-treatment of the cells with dex plus JIB-04 strongly increased cleavage of the apoptosis markers in comparison with dex alone or dex plus OG-L002 (Fig. 3c). This result confirmed that JIB-04 and dex increase apoptosis rather than simply inhibiting proliferation. Thus, in addition to increasing G9a methylation level, JIB-04 inhibitor also facilitates GC-induced cell death.

### JIB-04 inhibitor impacts G9a-regulated transcription

**Fig. 4:**
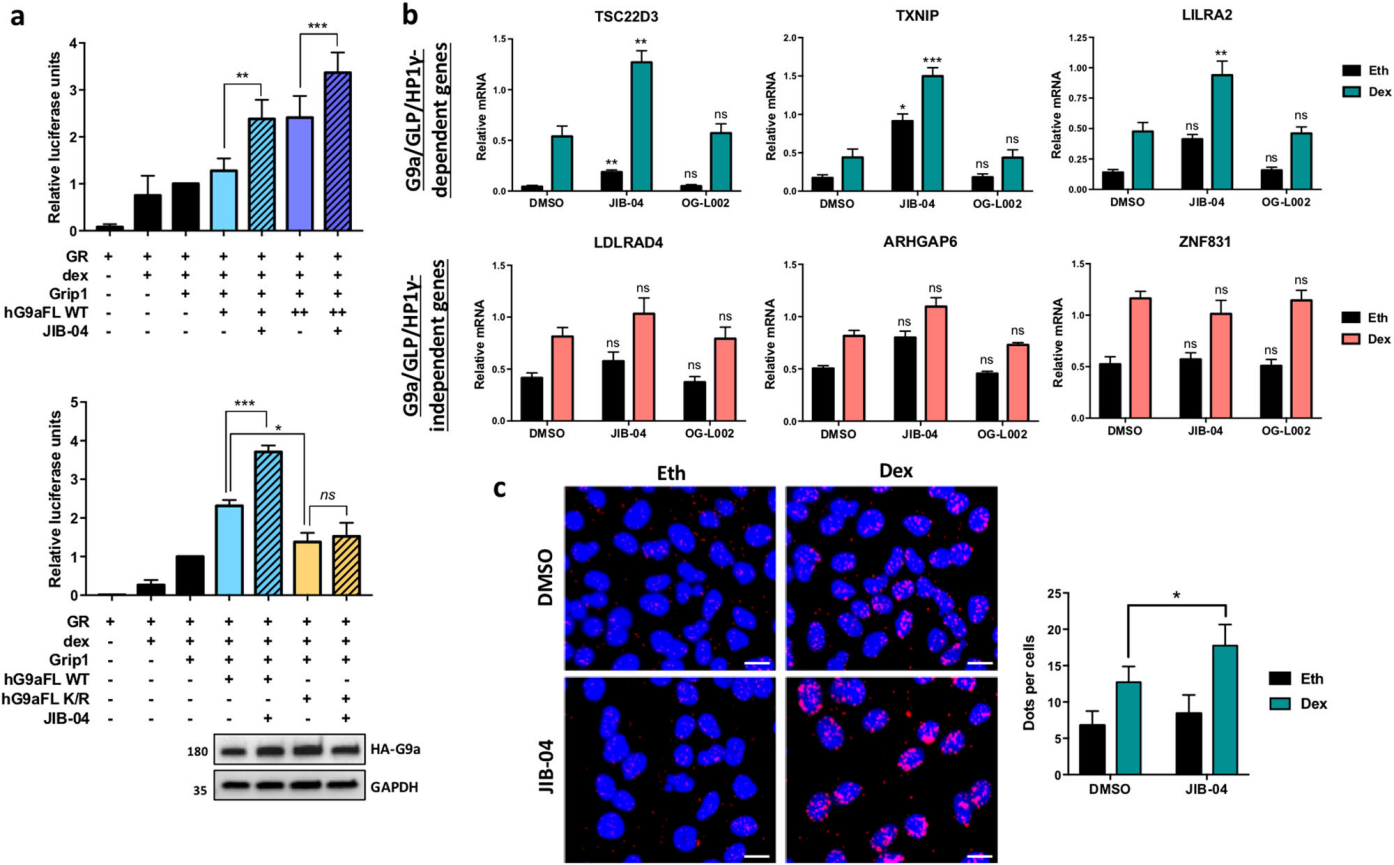
JIB-04 enhances G9a-regulated transcription. **a** CV-1 cells were transfected with MMTV-LUC reporter plasmid (200 ng) and plasmids encoding GR (1 ng), Grip1 (100 ng) and HA-labeled full-length (FL) hG9a wild type at 100 ng (+) or 250 ng (++) (upper panel) and HA-labeled full-length hG9a wild type or K185R mutant (250 ng) (lower panel). Cells were grown with 100 nM dex or the equivalent amount of ethanol, along with 0.5 μM JIB-04 or equivalent volume of DMSO for 24 h and assayed for luciferase activity. Relative luciferase units are normalized to sample 3 and represent mean ± SEM for five independent experiments, each with three biological replicates. The *p* value was calculated using a paired *t*-test: *p ≤ 0.05, **p ≤ 0.01, ***p ≤ 0.001, ns not significant. Whole-cell extracts were analyzed for G9a expression by immunoblot with indicated antibodies (lower panel). **b** Nalm6 cells were treated for 16 h with either JIB-04 at 0.5 μM, OG-L002 at 10 μM or DMSO. Then, cells were treated with 100 nM dex or ethanol for 8 h in addition to the previous drugs. mRNA levels for dex-regulated genes validated in Fig. 2b were measured by reverse transcriptase followed by qPCR and normalized to β-actin mRNA levels. Results shown are mean ± SEM for six independent experiments. The *p* value was calculated using a paired *t*-test between DMSO versus JIB-04 or OG-L002 samples: *p ≤ 0.05, **p ≤ 0.01, ***p ≤ 0.001, ns not significant. **c** To analyze interaction of endogenous GR and HP1γ by PLA, A549 cells were treated with 0.5 μM JIB-04 or the equivalent volume of vehicle DMSO for 24 h, then with the addition of 100 nM dex or the equivalent volume of vehicle ethanol (Eth) for 2 h. After cell fixation, PLA was performed with antibodies against GR and HP1γ. The detected interactions are indicated by red dots. The nuclei were counterstained with 4′,6-diamidino-2-phenylindole (DAPI; blue). The number of interactions detected by ImageJ analysis is shown as the mean ± SEM of three independent experiments. The *p* value was determined using a paired *t*-test: *p ≤ 0.05. Scale bar represents 10 μm

To explore the mechanism by which JIB-04 enhances GC-induced apoptosis of Nalm6 cells, we analyzed the impact on G9a coactivator function for GR and its target genes. Initially using a transient luciferase reporter gene that responds to dex, we observed that wild-type G9a acts cooperatively with another steroid receptor coactivator, GRIP1 (aka SRC-2 or NCoA2), in CV-1 cells (Fig. 4a) as previously demonstrated^13,19,20^. Treatment of the cells expressing GR, GRIP1 and wild-type hG9a with JIB-04 significantly increased luciferase activity (Fig. 4a). In contrast, an unmethylatable hG9a K/R mutant (lysine-185 methylation site changed to arginine), expressed at the same level as wild-type G9a, failed to enhance dex-induced expression of the reporter gene, and JIB-04 also had no effect on luciferase activity in cells expressing this mutant G9a (Fig. 4a, lower panels), indicating that the effect of JIB-04 on transcriptional activity requires G9a methylation.

Similar effects of JIB-04 were observed for endogenous GR target genes that require G9a and GLP. JIB-04 significantly increased dex-induced expression of three G9a/GLP/HP1γ-dependent genes, compared to the DMSO vehicle control (Fig. 4b, upper graphs), while OG-L002 had no effect on dex-regulated expression of these genes. In contrast, neither JIB-04 nor OG-L002 significantly affected the dex-regulated expression of three G9a/GLP/HP1γ-independent GR target genes (Fig. 4b, lower graphs). Thus, the effect of JIB-04 was restricted to GR target genes that are dependent on G9a and GLP, consistent with our conclusion that the increased dex-induced gene expression observed with JIB-04 is due to its specific enhancement of G9a/GLP methylation.

G9a/GLP automethylation creates a binding site for HP1γ which cooperates with G9a and GLP as a coactivator for GR^13^ (Figs. 1, 2). Since JIB-04 enhanced G9a methylation (Fig. 3a), we proposed that this should increase the interaction between G9a and HP1γ as well as the formation of a ternary GR/G9a/HP1γ complex (Fig. 2c). To test this hypothesis, we used the proximity ligation assay (PLA) in A549 lung adenocarcinoma cells, since the morphology of adherent cells provides a better experimental model than very small, spherical suspension cells like Nalm6 for viewing intracellular localization of the protein complexes detected by PLA. Dex treatment increased the observed proximity (indicating complex formation) between GR and HP1γ as previously described^13^, and JIB-04 significantly increased this interaction in the nucleus of dex-treated A549 cells (Fig. 4c), confirming that the enhanced G9a methylation caused by JIB-04 results in increased ternary GR-G9a-HP1γ complex formation which provides a mechanistic explanation for JIB-04 enhancement of dex-induced expression of G9a/GLP-dependent GR target genes.

### KDM4 family demethylates G9a

**Fig. 5:**
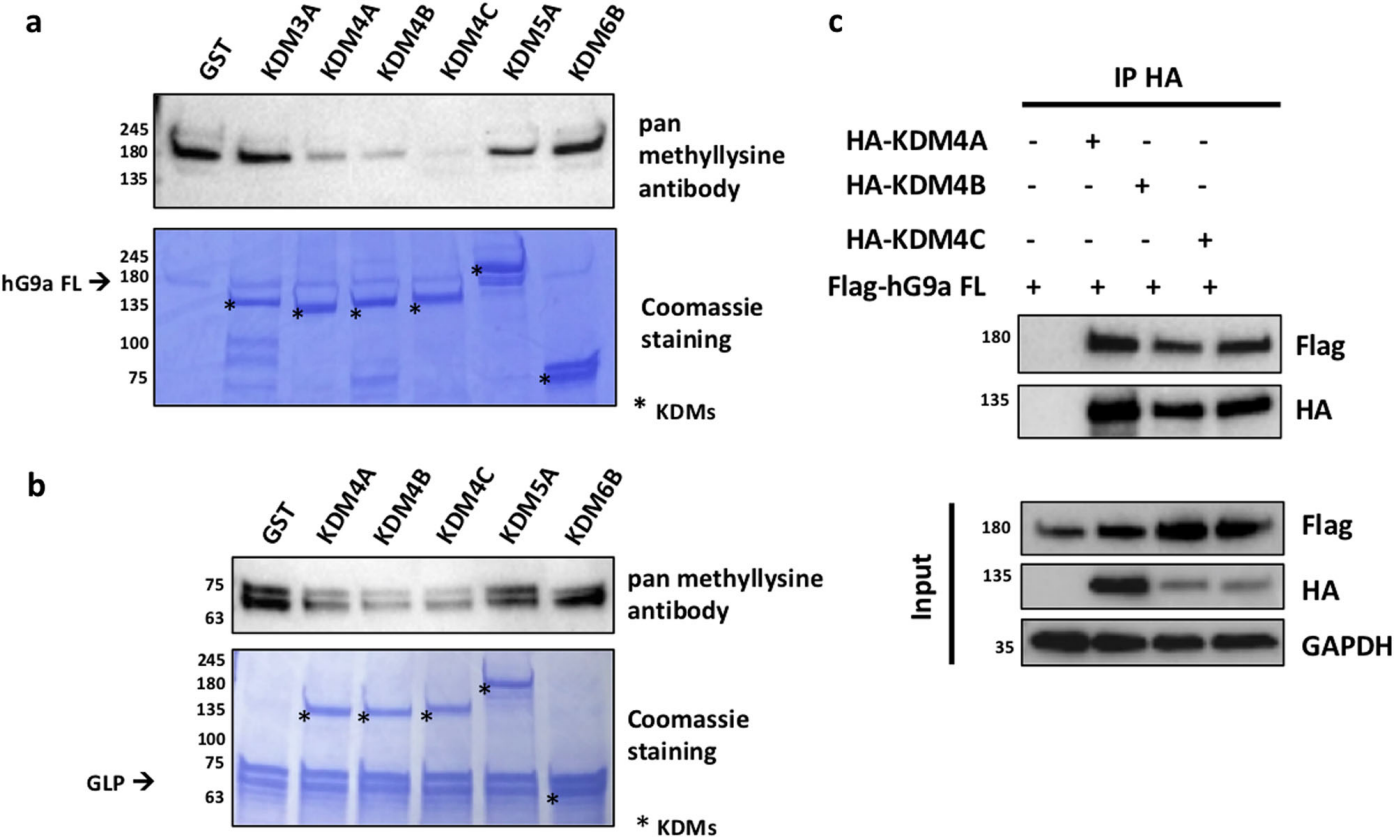
KDM4 family demethylates G9a and GLP. **a** Recombinant full-length (FL) hG9a was self-methylated with SAM, separated from residual SAM, then incubated with the indicated recombinant KDMs. Upper panel: Immunoblot with pan methyllysine antibody. Lower panel: After immunoblot analysis, the membrane was stained with Coomassie brilliant blue to detect quantities of assay protein components. Results shown are representative of three independent experiments. **b** Recombinant GST-hGLP N was methylated by GST-hGLP ΔN, separated from SAM, then incubated with the indicated recombinant KDMs. Upper panel: Immunoblot with pan methyllysine antibody. Lower panel: After detection, the membrane was Coomassie stained. Results shown are representative of three independent experiments. **c** Cos-7 cells were transfected with plasmids encoding flag-hG9a FL and HA-KDM4A, HA-KDM4B or HA-KDM4C. Lysates were immunoprecipitated with HA antibodies and analyzed by immunoblot using antibodies listed. Results shown are representative of three independent experiments

To begin identification of specific demethylases for G9a and GLP, we tested several recombinant enzymes from the JmjC family that have been shown to be inhibited by JIB-04 in cell-free reactions, including members of the KDM4, KDM5 and KDM6 families^35^. KDM4A, KDM4B and KDM4C reproducibly demethylated recombinant automethylated G9a (Fig. 5a) and GLP (Fig. 5b), while KDM3A, KDM5A and KDM6B had little or no activity. KDM4 family members also coimmunoprecipitated with G9a when these proteins were transiently over-expressed in Cos-7 cells (Fig. 5c).

### JIB-04 enhances dex repression of A549 cell migration

**Fig. 6:**
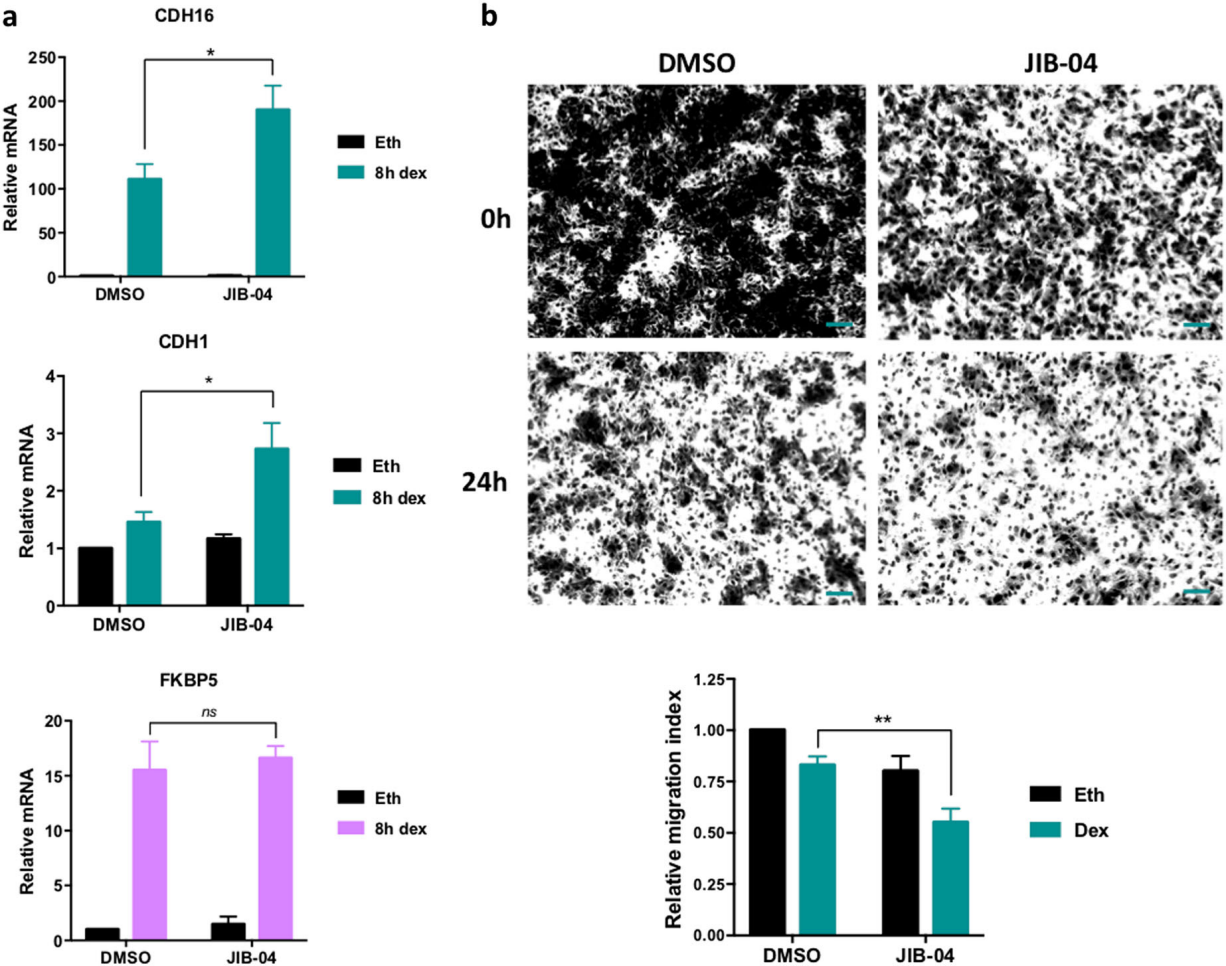
JIB-04 enhances dex repression of A549 cell migration. **a** A549 cells were treated for 16 h with either JIB-04 at 0.5 μM or DMSO. Then, cells were treated for 8 h with 100 nM dex or ethanol in addition to the previous drugs. mRNA levels for dex-regulated genes previously identified^13^ were measured by reverse transcriptase followed by qPCR and normalized to β-actin mRNA levels. Results shown are mean ± SEM for three independent experiments. The *p* value was calculated using a paired *t*-test between DMSO versus JIB-04 samples for dex-treated cells: *p ≤ 0.05, ns not significant. **b** A549 cell migration was analyzed using transwell migration assays. Migratory cells on the bottom of the polycarbonate membrane were stained. Representative images are shown (upper panel). Then, dye extracted from the cells was quantified at OD 560 nm. Relative migration in dex is shown as the mean ± SEM of three independent experiments (lower panel). The *p* value was determined using a paired *t*-test: **p ≤ 0.01. Scale bar represents 100 μm

To validate the role of G9a methylation and the effects of JIB-04 in an additional model system, we again used the A549 lung adenocarcinoma cell line to examine the dex regulation of cell migration. We previously demonstrated that G9a methylation is required for dex repression of A549 cell migration, because G9a is a required coactivator for dex to induce the expression of a specific subset of G9a/GLP-dependent GR target genes involved in cell migration, including E-cadherin (aka CDH1). We also demonstrated that G9a methylation is directly involved in the regulation of cell migration, as overexpression of a G9a K185R mutant (but not wild-type G9a) prevented dex repression of migration^13^. Here, we analyzed the effect of JIB-04 inhibitor on A549 cell migration and key genes previously identified. We observed that JIB-04 further enhanced dex-induced expression of two G9a/GLP-dependent GR target genes, E-cadherin/CDH1 and CDH16, but had no effect on dex-induced expression of a G9a/GLP-independent gene, FKBP5 (Fig. 6a). Corresponding to the increase in dex-induced E-cadherin expression caused by JIB-04, the repressive effect of dex on A549 cell migration was also enhanced by JIB-04 (Fig. 6b).

## Discussion

Few good treatment options are currently available for childhood ALL patients who relapse after initial chemotherapy. Chimeric antigen receptor (CAR) T cells represent a new potential option for relapsed patients with B-ALL, but even for this new and promising genre of treatment, escape mechanisms are common^36^. For relapsed patients with T-ALL, the options are even more limited^37^. Chemoresistance, particularly to GC, is usually a prime characteristic of relapse^1,3,38^. Here we provide the basis for a new potential treatment option, a protein lysine demethylase inhibitor that selectively enhances GC-induced cell death in leukemia cells. JIB-04 selectively enhanced GC activation of a subset of GR target genes (Fig. 4b) that includes genes encoding proteins that help to mediate GC-induced death of lymphoid cells (Fig. 2a); the inhibitor also enhanced GC-induced death of the B-ALL cell line Nalm6 (Fig. 3). Our findings provide the impetus for preclinical testing of this class of protein lysine demethylase. This method to enhance sensitivity to GC-induced cell death may be relevant to other types of lymphoid-derived blood cancers that are normally treated with GC, and for which GC resistance is also a common characteristic of relapse.

The selective effect of the JIB-04 protein lysine demethylase inhibitor on GC-regulated genes that require G9a, GLP and HP1γ as coactivators adds further support for the molecular model we have proposed (Fig. 2c). JIB-04 increases the methylation level of G9a (Fig. 3a), which enhances its interaction with HP1γ and its ability to form a ternary GR/G9a/HP1γ complex (Fig. 4c) that is assembled on GR binding sites of a subset of GR target genes, specifically those that require G9a, GLP and HP1γ for their GC-induced expression^13^. We further demonstrated by genome-wide RNA-seq analysis that the great majority of G9a/GLP-dependent GR target genes also require HP1γ for their GC-regulated expression, while the great majority of G9a/GLP-independent GR target genes do not require HP1γ (Fig. 1d, e), thus validating our proposed molecular model on a genome-wide basis. Since this molecular model of selective regulation of GR target genes by G9a, GLP and HP1γ is operational in cell lines representing two very different cell types—A549 lung adenocarcinoma cells and Nalm6 B-ALL cells—it appears likely that it will be operational in many if not all cell types.

In addition to G9a and GLP, JIB-04 also inhibits the demethylation of H3K9 (Fig. 3a) and presumably other methylated proteins as well. It is thus appropriate to question whether the enhancement of GC-induced expression of GR target genes and GC-induced Nalm6 cell death by JIB-04 is due to increased methylation of G9a/GLP or of other protein substrates, especially since H3K9 methylation is well known to regulate transcription. We provide several experimental results that point toward the increase in G9a/GLP methylation as the critical factor. First, the effect of JIB-04 on transcription is selective: JIB-04 enhanced GC-induced expression of G9a/GLP-dependent genes but not G9a/GLP-independent genes (Fig. 4b). Second, in a transient reporter gene assay, JIB-04 enhanced the coactivator activity of wild-type G9a but had no effect on a G9a mutant that cannot be methylated (Fig. 4a). Third, increased H3K9 methylation on enhancer elements such as GR binding sites would be expected to reduce transcription on a global scale, but JIB-04 *selectively enhanced* GC-induced expression of G9a/GLP-dependent GR target genes and had no effect on GC-induced expression of G9a/GLP-independent target genes (Fig. 4b). These results support the increase in G9a/GLP methylation as the molecular mechanism for the selective effect of JIB-04 on a subset of GR target genes.

Multiple demethylase enzymes are generally expressed in any given cell type, and our in vitro assays with recombinant enzymes and substrates indicated that several different JmjC family enzymes are capable of demethylating G9a and GLP (Fig. 5). Three KDM4 isoforms, which are encoded by different genes, were quite active against G9a and GLP, and KDM5A was also modestly active against G9a. Our attempts to identify the specific demethylase(s) responsible for G9a/GLP demethylation in Nalm6 cells have been unsuccessful, since depletion of individual enzymes, and even combinations of two or three enzymes, generally had no effect on G9a/GLP methylation status and coactivator function. Therefore, it is likely that the effect of JIB-04 on G9a/GLP methylation status and coactivator function is due to its inhibition of multiple JmjC protein lysine demethylase enzymes, including several KDM4 isoforms and KDM5A^35^. We speculate that the reason for the specificity of JIB-04 against a subset of JmjC enzymes may be due to specific physical characteristics of the active sites of these enzymes that dictate similar substrate specificities (e.g. against H3K9 methylation and G9a/GLP methylation). Indeed, the methylation site sequences of H3K9 (ARKS) and G9a/GLP (ARKT) are almost identical.

The coregulator actions of G9a and GLP are quite gene specific, helping GR to activate some genes and repress other genes while being irrelevant for GC-regulated expression of many GR target genes (Fig. 1). This gene specificity is typical of GR coregulators^9,11,12^ and is apparently a product of evolutionary engineering to allow the many physiological pathways controlled by GC to be somewhat independently modulated by regulation of coregulators. This is suggested by findings that the sets of GC-regulated genes that require a specific coregulator are enriched for specific GC-regulated physiological pathways^12–14^. In Nalm6 cells, pathway analysis of G9a/GLP-dependent GR target genes indicated enrichment for genes involved in cell proliferation and cell death (Fig. 2a, table S1), while in A549 cells G9a/GLP-dependent GR target genes were enriched for genes involved in cell migration^13^. Thus, regulation of specific coregulators, by regulation of their protein levels or their activity, would be expected to modulate a subset of the physiological pathways controlled by GC. In the case of G9a and GLP, such regulation is accomplished by post-translational modifications, i.e., automethylation and phosphorylation. The gene-specific actions of coregulators makes them potentially attractive targets for selective manipulation of the actions of a particular transcription factor. In our case, the use of JIB-04 demethylase inhibitor, by enhancing the coactivator function of G9a and GLP, selectively enhances the effect of GC on leukemia cell death, while apparently having no effect on genes involved in regulating other physiological pathways. This selective enhancement of specific GC-regulated physiological pathways may also make it possible to limit the dosing of GC and correspondingly reduce deleterious side effects caused by extended high-dose GC treatments. Testing of preclinical tumor models will be required to determine the potential of these inhibitors for human clinical trials.

## Electronic supplementary material

Supplemental figures
