# Supplementary material for: Increasing G9a automethylation sensitizes B acute lymphoblastic leukemia cells to glucocorticoid-induced death
Source: Cell Death Dis. 2018 Oct 10;9(10):1038. doi: 10.1038/s41419-018-1110-z (PMC6180122; doi:10.1038/s41419-018-1110-z)
Supplement: Supplementary file 1 — Supplemental figures [file 41419_2018_1110_MOESM1_ESM.docx]

**SUPPLEMENTAL INFORMATION**

**SUPPLEMENTAL FIGURE LEGENDS**

**
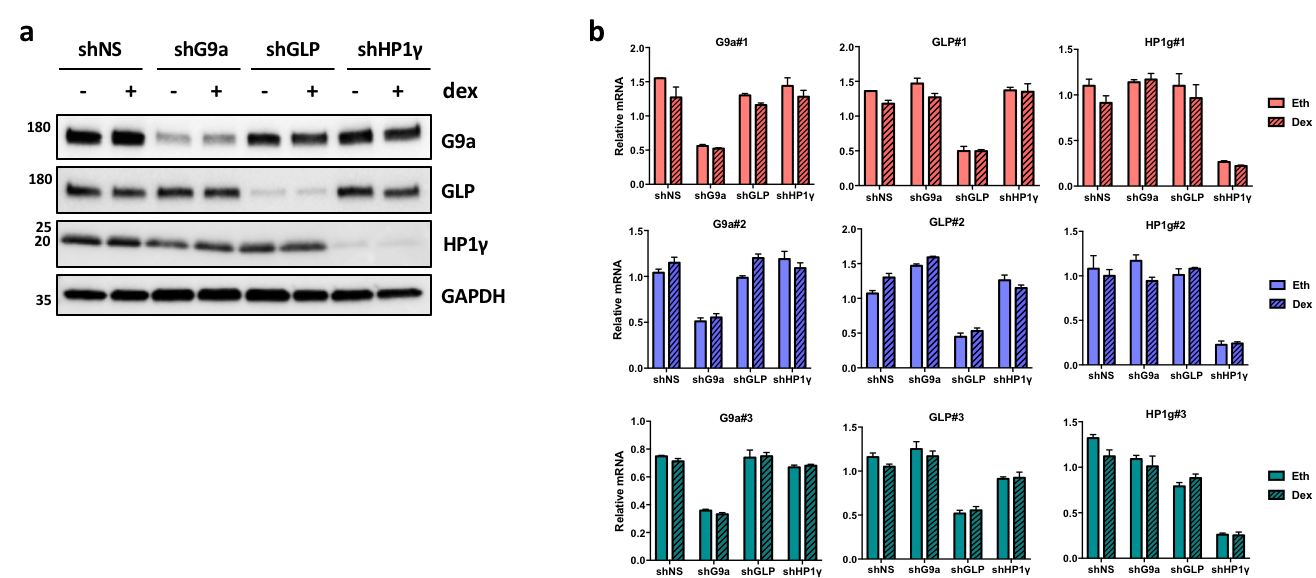
**

**Figure S1 :**

NALM-6 cells used in the RNA seq experiment were transduced with lentivirus encoding shRNA against a nonspecific sequence (shNS), shG9a, shGLP or shHP1γ, and then analyzed by immunoblot for the indicated proteins (**a**) and by RT-qPCR for the indicated mRNAs (**b**) after treatment with 100 nM dex or the equivalent volume of ethanol (Eth) for 8 h. RT-qPCR was performed on total RNA. mRNA levels are shown relative to β-actin mRNA for each of the three replicates and are mean ± SD from three technical qPCR replicates.


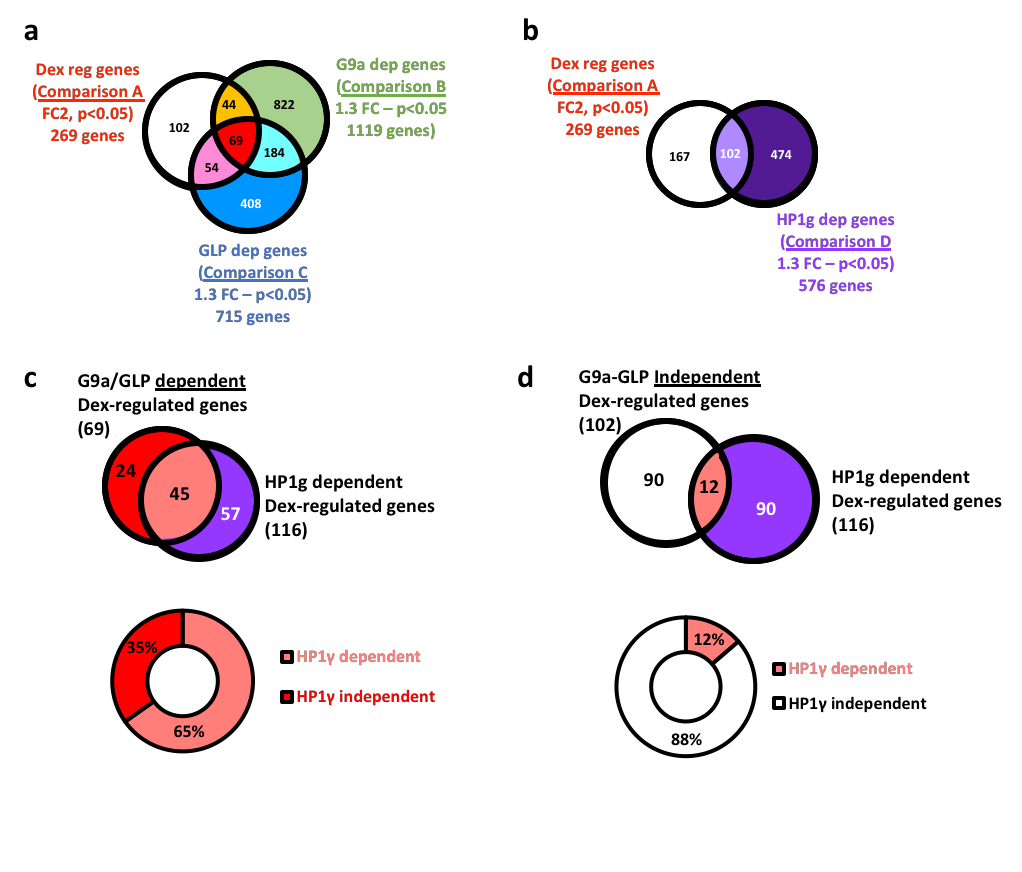


**Figure S2**

The bioinformatic analysis performed in Fig. 1 was repeated with more stringent parameters to identify the sets of genes dependent on G9a, GLP, and HP1γ (1.3 fold change cutoff for the effects of coregulator depletion instead of no fold-change cutoff, along with adjusted p ≤ 0.05). (**a**) White Venn diagram represents the dex-regulated genes for cells expressing shNS and treated with 100 nM dex for 8 h compared with ethanol (Comparison A from Fig. 1a, p-value ≤ 0.05 and at least 2-fold increase or decrease); Green Venn diagram, G9a-dependent genes (Comparison B from Fig. 1a, p-value ≤ 0.05 and at least 1.3-fold increase or decrease); Blue Venn diagram, GLP-dependent genes (Comparison C from Fig. 1a, p-value ≤ 0.05 and at least 1.3-fold increase or decrease). Overlap areas indicate the number of genes shared among sets. (**b**) White Venn diagram represents the dex-regulated genes for cells transfected with shNS and treated with 100 nM dex for 8 h compared with ethanol (Comparison A from Fig. 1a, p-value ≤ 0.05 and at least 2-fold increase or decrease); Purple Venn diagram, HP1γ-dependent genes (Comparison D from Fig. 1a, p-value ≤ 0.05 and at least 1.3-fold increase or decrease). Overlap area indicates the number of genes shared among sets. (**c**) Red Venn diagram represents the dex-regulated genes that are dependent on both G9a and GLP, identified in the central red overlap area of Fig. S2a; Purple Venn diagram, dex-regulated genes that are dependent on HP1γ, identified in the overlap area of Fig. S2b. The percentages of G9a/GLP-dependent genes that are HP1γ-dependent or HP1γ-independent are represented in the donut graph. (**d**) White Venn diagram represents the dex-regulated genes that are independent of both G9a and GLP, identified in the white sector of Fig. S2a; Purple Venn diagram, dex-regulated genes that are dependent on HP1γ, identified in the overlap area of Fig. S2b. The percentages of G9a/GLP-independent genes that are HP1γ-dependent or HP1γ-independent are represented in the donut graph.

| **Category** | **p-value** | **Molecules** |
| --- | --- | --- |
| Cell Cycle | 1.13E-06-5.38E-03 | SOCS1,PER1,TXNIP,BTG1,YBX3,KLF13,SERTAD1,H2AFB3 (includes others),ID3,TSC22D3,MYT1,NFKBIA,BTG2,SOCS2,IL1B,ZC3H12D,ESR1,MXI1 |
| Gene Expression | 1.76E-06-5.19E-03 | SOCS1,PER1,TFAP2C,TXNIP,BTG1,YBX3,KLF13,SERTAD1,ID3,TSC22D3,MYT1,NFKBIA,BTG2,SOCS2,STAB1,IL1B,ESR1,MXI1 |
| Cellular Development | 3.15E-06-6.49E-03 | SOCS1,CLN8,KCNJ2,DISP3,UBASH3B,TSC22D3,NFKBIA,LILRA2,MCAM,HIST1H4L,SOCS2,FKBP5,MXI1,BTNL9,TFAP2C,TXNIP,CD69,BTG1,KIF26A,SERTAD1,H2AFB3 (includes others),ID3,MYT1,RASSF4,BTG2,IL1B,ZC3H12D,ESR1 |
| Cellular Growth and Proliferation | 3.15E-06-6.49E-03 | PER1,SOCS1,LOXL4,KCNJ2,UBASH3B,TSC22D3,NFKBIA,LILRA2,MCAM,HIST1H4L,SOCS2,FKBP5,MXI1,BTNL9,TFAP2C,TXNIP,CD69,BTG1,KIF26A,SERTAD1,ID3,H2AFB3 (includes others),GP5,MYT1,RASSF4,BTG2,IL1B,ZC3H12D,ESR1 |
| Cellular Function and Maintenance | 4.66E-06-5.19E-03 | SOCS1,KCNJ2,TXNIP,CD69,UBASH3B,SLC20A2,ID3,TSC22D3,MYT1,NFKBIA,MCAM,SOCS2,IL1B,ESR1 |
| Cell Death and Survival | 2.81E-05-6.41E-03 | SOCS1,PER1,TFAP2C,CLN8,KCNK3,TXNIP,CD69,BTG1,YBX3,TDRD9,KLF13,ID3,H2AFB3 (includes others),TSC22D3,NFKBIA,MCAM,RASSF4,BTG2,SOCS2,IL1B,FKBP5,  ESR1,MXI1 |
| Cell Morphology | 6.59E-05-5.19E-03 | SOCS1,NFKBIA,KCNJ2,TXNIP,MCAM,IL1B,KLF13,ID3,ESR1,MXI1 |
| Cellular Compromise | 6.59E-05-5.59E-03 | SOCS1,NFKBIA,MCAM,BTG2,IL1B |
| Cell-To-Cell Signaling and Interaction | 9.88E-05-5.59E-03 | PER1,SOCS1,KCNJ2,TXNIP,CD69,ID3,TSC22D3,GP5,NFKBIA,LILRA2,MCAM,SOCS2,IL1B,STAB1,ESR1,MXI1 |
| Molecular Transport | 1.38E-04-5.19E-03 | PER1,NFKBIA,KCNK3,KCNJ2,TXNIP,IL1B,ESR1,TSC22D3 |
| Small Molecule Biochemistry | 1.38E-04-6.11E-03 | PER1,SOCS1,NFKBIA,IL1B,ESR1,TSC22D3 |
| Cellular Movement | 1.7E-04-5.91E-03 | SOCS1,TFAP2C,NFKBIA,MCAM,CD69,BTG2,SOCS2,STAB1,IL1B,ID3,ESR1 |
| Lipid Metabolism | 5.91E-04-5.19E-03 | PER1,NFKBIA,IL1B,ESR1,TSC22D3 |
| Protein Trafficking | 5.91E-04-2.18E-03 | NFKBIA,KCNJ2,HIST1H4L,IL1B,ESR1,MXI1 |
| Cellular Assembly and Organization | 1.1E-03-5.85E-03 | CYTH3,SOCS1,KCNJ2,BTG2,STAB1,IL1B,ESR1 |
| Drug Metabolism | 1.1E-03-2.6E-03 | IL1B,ESR1,TSC22D3 |
| Carbohydrate Metabolism | 2.6E-03-5.19E-03 | SOCS1,NFKBIA,IL1B |
| Energy Production | 2.6E-03-2.6E-03 | IL1B |
| Cell Signaling | 2.91E-03-5.19E-03 | SOCS1,PER1,NFKBIA,SOCS2,IL1B,ESR1 |
| Protein Synthesis | 4.59E-03-4.59E-03 | SOCS1,NFKBIA,CD69,ID3,ESR1 |
| Nucleic Acid Metabolism | 5.19E-03-5.19E-03 | IL1B,ESR1 |
| Post-Translational Modification | 5.34E-03-5.34E-03 | BTG2,BTG1 |

**Table S1**

The enriched categories from the analysis in Fig. 2a are shown, along with the p-value for the enrichment and the identities of genes included in each category (Ingenuity Pathway Analysis).
